# Retiring the Term “Weighted Mean Difference” in Contemporary Evidence Synthesis

**DOI:** 10.1002/cesm.70051

**Published:** 2025-09-11

**Authors:** Lifeng Lin, Xing Xing, Wenshan Han, Jiayi Tong

**Affiliations:** ^1^ Department of Epidemiology and Biostatistics University of Arizona University of Arizona Tucson Arizona USA; ^2^ Department of Biostatistics Johns Hopkins Bloomberg School of Public Health Baltimore Maryland USA; ^3^ Department of Population and Community Health University of North Texas Health Science Center Fort Worth Texas USA

## 1 Background

Evidence synthesis frequently involves quantitative analyses of continuous outcomes. A cross‐sectional study examining Cochrane systematic reviews identified 6672 out of 22,453 meta‐analyses (29.7%) involved continuous outcomes [1]. The primary effect measures employed in meta‐analyses of continuous outcomes are the mean difference (MD) and standardized mean difference (SMD) [2]. The MD is appropriately applied when all included studies measure outcomes using identical scales (e.g., body weight in kilograms). In contrast, the SMD serves as a solution when studies utilize different measurement scales (e.g., varied questionnaire scoring methods). Although alternative measures (e.g., the ratio of means) exist [3], their application remains relatively infrequent.

Despite this conceptual clarity, the term “weighted mean difference” (WMD) appears frequently in the systematic review literature [4], which can lead to confusion about its relationship to the MD. In this article, we first clarify the distinction between MD and WMD, then describe the historical factors underlying the term's adoption and persistence, discuss why contemporary methods render it unnecessary, illustrate examples of misuse, and conclude with practical recommendations for clearer reporting.

## 2 What are MD and WMD

The MD represents the straightforward difference between group means (e.g., intervention vs. control) for a continuous outcome. Although the true MD value relates to unknown population‐level differences, practical research relies on sample estimates from individual studies. Meta‐analysis systematically synthesizes these study‐level MD estimates to derive an overall summary effect across studies.

The term WMD emerged historically to emphasize the weighted averaging process of meta‐analyses, wherein each study contributes a sample MD weighted by its statistical precision (i.e., inverse variance) [5]. Typically, larger studies with smaller variances or narrower confidence intervals are assigned greater weights. Traditional meta‐analytical methods, performed through either fixed‐effect (also known as common‐effect) or random‐effects models, follow this inverse‐variance weighting principle. Under fixed‐effect models, study weights directly reflect the inverse of their variances, whereas random‐effects models incorporate both within‐study and between‐study variances.

## 3 History of How WMD Has Evolved

**Figure 1:**
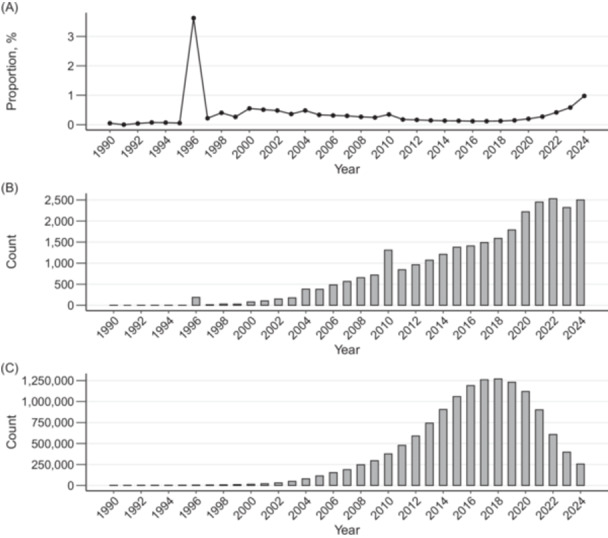
(A) Trend in the proportion of publications mentioning both systematic reviews and weighted mean differences among all publications mentioning systematic reviews, from 1990 to 2024. (B) Yearly counts of publications mentioning both systematic reviews and weighted mean differences. (C) Yearly counts of publications mentioning systematic reviews.

To contextualize the widespread adoption of WMD, we conducted a brief literature search using Google Scholar on June 12, 2025. Using exact‐phrase queries in quotation marks, for each calendar year from 1990 to 2024, we recorded the counts for “weighted mean difference” AND “systematic review” and separately for “systematic review,” then calculated the yearly proportion (Figure 1). Google Scholar indexes titles, abstracts, and, when available, full texts, so counts reflect occurrences anywhere in the indexed record, and these counts are approximate. We did not screen individual records for correct versus incorrect usage because our objective was to describe the prevalence of terminology rather than to quantify misuse. We therefore documented the evolution of usage over time in the proportions reported in Figure 1.

This analysis revealed an observable increase in WMD usage around 1996, closely following the establishment of the *Cochrane Database of Systematic Reviews* (CDSR) in April 1995. The influential role of Cochrane reviews likely contributed greatly to disseminating this terminology. Chapter 6.5 of the latest Cochrane Handbook [6] confirms the prevalence of the term “weighted mean difference” in early editions of CDSR, with such cautionary notes appearing in handbook versions since at least 2008: “Analyses based on this effect measure have historically been termed [WMD] analyses in the [CDSR]. This name is potentially confusing: although the meta‐analysis computes a weighted average of these differences in means, no weighting is involved in the calculation of a statistical summary of a single study. Furthermore, all meta‐analyses involve a weighted combination of estimates, yet we don't use the word ‘weighted’ when referring to other methods.”

Another plausible factor contributing to the continued use of the term is the citation of Andrade's statement in 2020 that “the pooled MD is more accurately described as a weighted mean difference or WMD.” [7] While this interpretation is not technically incorrect in describing the statistical process behind meta‐analytic pooling, it may inadvertently encourage broader or careless use of the term WMD.

Despite existing notes on WMD in the literature, Figure 1 illustrates the continued widespread use of WMD. Specifically, while the total number of systematic review publications increased until peaking around 2018 and then declined (Figure 1C), both the number and proportion of publications mentioning WMD continued to rise through 2024 (Figure 1A,B). Although the term is not misused in all instances, this trend suggests that existing cautions have had limited impact and underscores the value of clearer terminology. These historical and descriptive observations motivate a focus on current analytic practice and terminology, as discussed next.

## 4 Contemporary Evidence Synthesis Does Not Justify Using WMD

The explicit emphasis on weighting inherent to the term WMD can be misleading because weighting is fundamental to conventional meta‐analytical methods, regardless of the outcome type (continuous, binary, time‐to‐event, etc.). Nevertheless, analogous terms such as “weighted odds ratio” or “weighted hazard ratio” are rarely used. Hence, more general terms such as “pooled MD,” “combined MD,” “overall MD,” or “meta‐analytical MD” may be more appropriate and consistent.

Moreover, contemporary methodological advancements in evidence synthesis frequently extend beyond traditional inverse‐variance weighting. Modern meta‐analyses, including pairwise and network applications, are often fit as one‐stage generalized linear mixed or Bayesian hierarchical models in which treatment effects are estimated jointly from the likelihood [8, 9, 10]. In these models, precision is incorporated through the model structure rather than through explicit study‐specific inverse‐variance weights. Consequently, when outcome scales are identical, the pooled estimate is more clearly reported as a pooled MD or another clear descriptor, such as meta‐analytic MD; the term WMD is unnecessary and may suggest a distinct effect measure. Imprecise usage nonetheless persists in current literature, as illustrated below.

## 5 Potential Misuse of WMD

Critically, MD specifically pertains to individual study outcomes, while WMD exclusively represents the meta‐analytical synthesis. Despite this clear distinction, some systematic reviews incorrectly label individual study effects as WMD [11, 12, 13, 14]. For example, a systematic review published recently in *JAMA* inaccurately reported “pooled weighted mean differences” for systolic and diastolic blood pressures between screening and control groups [11]. Here, the pooled MD inherently indicates weighting, making the addition of “weighted” redundant and misleading. Moreover, a recent article in the *American Journal of Ophthalmology* captions a forest plot as “weighted mean differences (WMD) … across each study.” [12] Another applied paper captions a forest plot as “WMD and 95% CI,” both implying study‐level WMDs [13]. In addition, a methods book chapter explicitly states, “Table 3.4 presents the WMD and the 95% confidence interval for each study.” [14] Such misuse persists in systematic reviews over time, including many published in various high‐impact journals [15].

Labeling study‐level effects as “WMD” can blur the distinction between a study's MD and the pooled meta‐analytic estimate. For instance, a figure caption that states “WMD across each study” may suggest that each study yields a WMD rather than an MD, which can confuse evidence users about what is being pooled. Clearer labeling (e.g., “MD per study” with a “pooled MD”) reduces this risk and improves interpretability.

## 6 Conclusions

This article underscores the potential inappropriateness of the term WMD, particularly its incorrect application to individual studies in evidence synthesis. Originating largely from early practices in Cochrane systematic reviews, WMD no longer aligns with contemporary methodological needs and rigor. Consequently, we recommend retiring the term WMD and adopting clearer terminology, using MD for study‐level effects and pooled MD or meta‐analytic MD for the synthesized estimate, to promote clearer, methodologically sound communication.

## Author Contributions

**Lifeng Lin:** conceptualization, funding acquisition, investigation, writing – original draft, visualization, writing – review and editing. **Xing Xing:** investigation, writing – review and editing. **Wenshan Han:** data curation, writing – review and editing, visualization. **Jiayi Tong:** conceptualization, writing – review and editing.

## Conflicts of Interest

The authors declare no conflicts of interest.

## 1 Peer Review

The peer review history for this article is available at https://www.webofscience.com/api/gateway/wos/peer-review/10.1002/cesm.70051.

## Data Availability

Data sharing is not applicable to this article as no data sets were generated or analyzed during the current study.
